# Supplementary material for: Crystal structure of Leishmania donovani glucose 6-phosphate dehydrogenase reveals a unique N-terminal domain
Source: Commun Biol. 2022 Dec 9;5:1353. doi: 10.1038/s42003-022-04307-7 (PMC9734377; doi:10.1038/s42003-022-04307-7)
Supplement: Supplementary file 2 — Supplementary Information [file 42003_2022_4307_MOESM2_ESM.pdf]

Supplementary Information

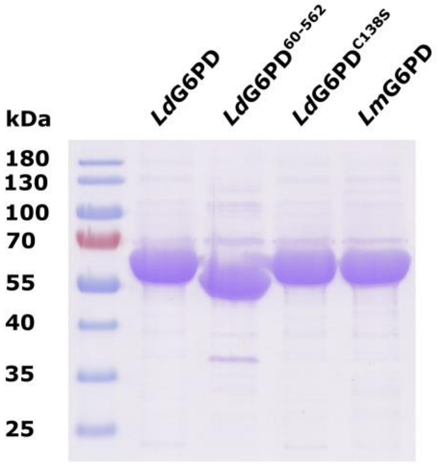

Fig. S1 Coomassie-stained SDS-PAGE gel (12% polyacrylamide) of purified *Ld*G6PD wt, *Ld*G6PD$^{60\text{-}562}$, *Ld*G6PD^{C138S}, and *Lm*G6PD wt. N-terminally His-tagged proteins were purified via Ni-NTA affinity chromatography followed by size exclusion chromatography. Left: Prestained protein ladder No. 26616 (Thermo Scientific, Dreieich, Germany). (*Ld*G6PD wt / *Ld*G6PD^{C138S}: 66.7 kDa; *Ld*G6PD$^{60\text{-}562}$: 60.3 kDa; *Lm*G6PD wt: 66.8 kDa)

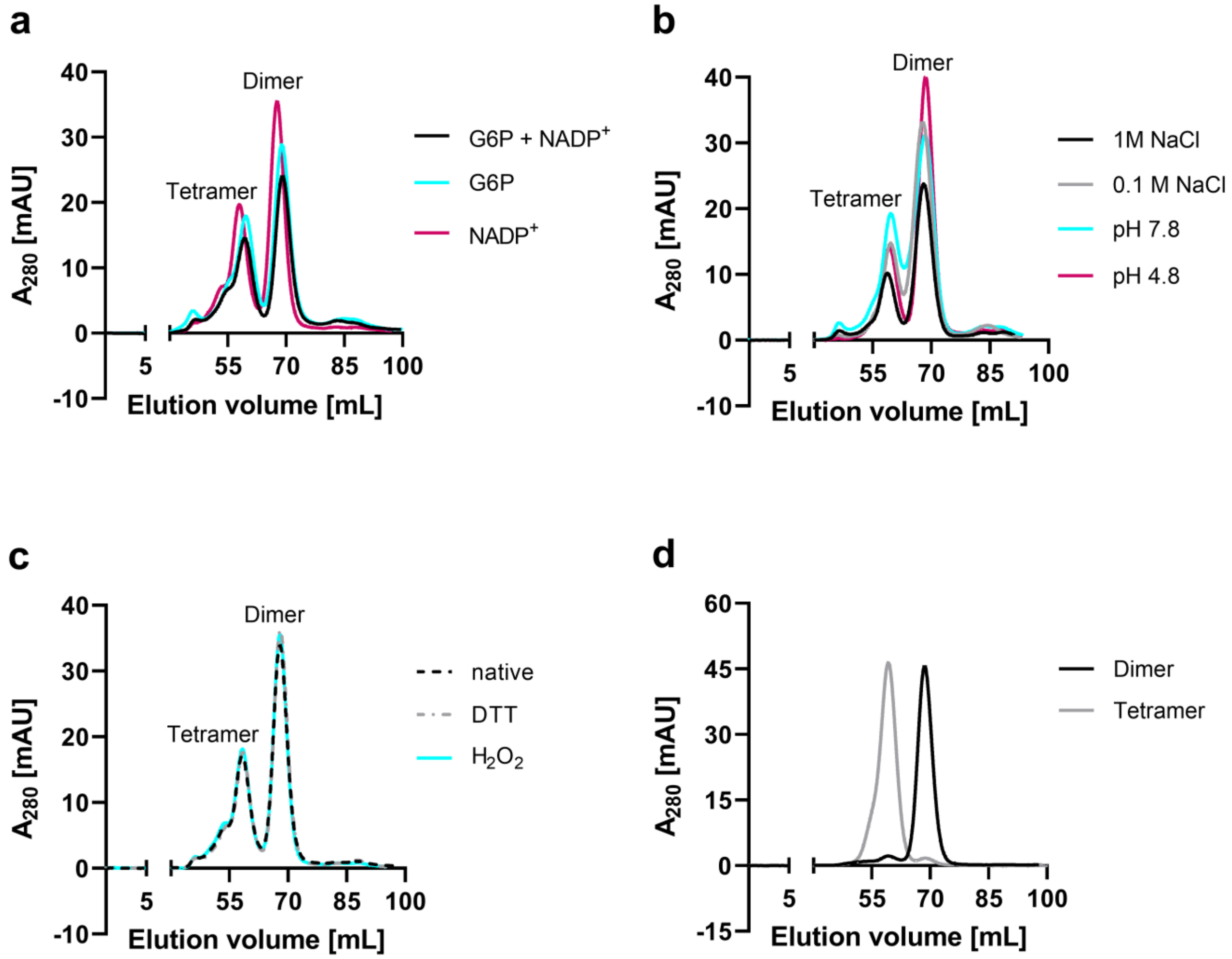

Fig. S2 SEC analysis of recombinant *Ld*G6PD wt. Full-length G6PD was gel filtrated on a HiLoad 16/60 Superdex 200 column pre-equilibrated in buffer A (500 mM NaCl, 50 mM Tris, pH 7.8), in the presence or absence of different ligands (**a, c, d**) or pre-equilibrated in buffer A with NaCl concentrations (0.1 mM, 1 mM) or pH values (pH 4.8, pH 7.8) different from the standard buffer A (**b**). **a** SEC profiles of *Ld*G6PD wt in the presence of substrates G6P + $NADP^+$ (black, rv 1. peak = 59 mL = 276 kDa, 2. peak = 69 mL = 122 kDa), G6P (blue, 1. peak = 59 mL = 276 kDa, 2. peak = 69 mL = 122 kDa) and $NADP^+$ (red, rv 1. peak = 58 mL = 300 kDa, rv 2. peak = 68 mL = 133 kDa) display two peaks with stable elution patterns equivalent to a dimer and a tetramer. **b** SEC of *Ld*G6PD wt in the presence of 1 M NaCl (black), 0.1 mM NaCl (grey), pH 7.8 (blue) and pH 4.8 (red) revealed elution patterns with two peaks equivalent to a tetramer (rv = 59 mL = 276 kDa) and a dimer (rv = 68 mL = 133 kDa). **c** SEC of *Ld*G6PD wt under native conditions (black-dotted), in the presence of DTT (grey-dotted) and H_2O_2 (blue, rv 1. peak = 57 mL = 325 kDa, rv 2. peak = 67 mL = 144 kDa) revealed identical elution patterns with two peaks equivalent to a tetramer (rv = 58 mL = 300 kDa) and a dimer (rv = 68 mL = 133 kDa). **d** Separately pooled *Ld*G6PD wt dimer (black, rv = 68 mL = 133 kDa) and tetramer fraction (grey, rv = 59 mL = 276 kDa) remain in their original conformation when reapplied to the SEC column. Representative chromatograms ($n \geq 2$) are shown for each condition.

Table S1 Crystallographic data

		*Ld*G6PD wt				*Ld*G6PD[60-562]	*Ld*G6PD[C138S]	
**PDB code**		7ZHT	7ZHU	7ZHV	7ZHW	7ZHX	7ZHY	7ZHZ
**Crystallized SEC fraction**		Dimer	Dimer	Dimer	Tetramer	Dimer	Dimer	Dimer
**Space group**		P2	C2	C2	C2	C222	C2	C2
**Molecules per AU**		4	2	2	2	1	2	2
**Oligomerization per AU**		2 Dimers	Dimer	Dimer	Dimer	Monomer	Dimer	Dimer
**Tetramer constructed via crystallographic symmetry operations**		*Ld*G6PD	*Ld*G6PD	*Ld*G6PD	*Ld*G6PD	*T. cruzi*	*Ld*G6PD	*Ld*G6PD
**Co-crystallization**		G6P	G6P, $NADP^+$	G6P	G6P, $NADP^+$	G6P, $NADP^+$	G6P, $NADP^+$	G6P, $NADP^+$
	Monomer							
**Visible ligands**	A	-	NADP(H)	-	NADP(H)	NADP(H)	NADP(H)	NADP(H)
	B	-	NADP(H)	G6P	G6P, NADP(H)	NADP(H)	NADP(H)	G6P, NADP(H)
**Loop between N- and core domain (~D50-K64)** *Missing residues are listed*	A	52-63	50-55	complete	complete	deleted	52-64	57-63
	B	49-62	51-54	52-62	52-54	= A	52-63	55-67
**Loop involved in interaction with the N-domain (~T471-D482)** *Missing residues are listed*	A	complete	476-479	472-481	473-479	472-479	476-479	473-479
	B	474-477	complete	473-479	473-479	= A	complete	478-479

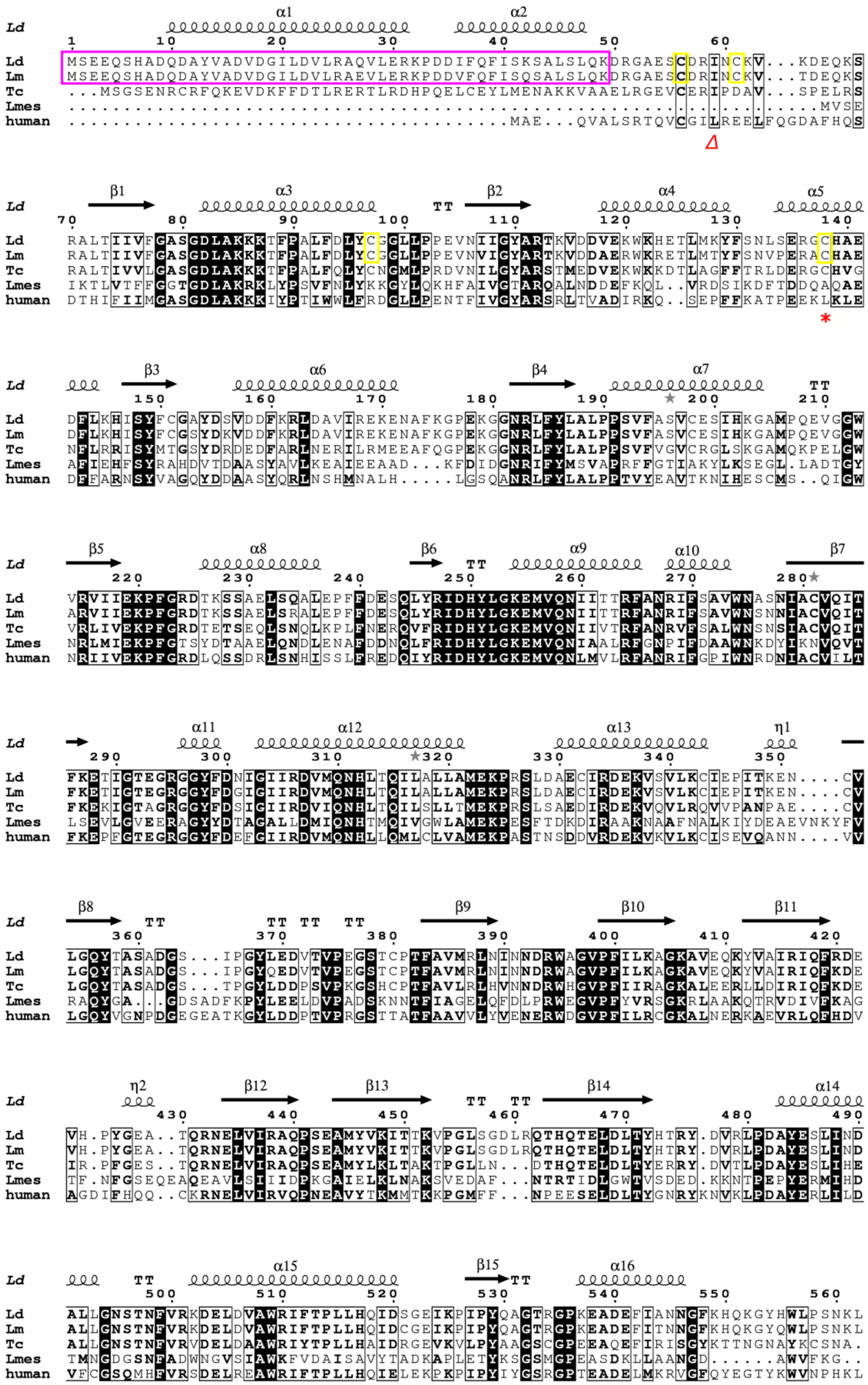
Ld
Lm
Tc
Lmes
human

Fig. S3 Structure-based multiple sequence alignment of G6PDs from various species. Shown G6PDH sequences correspond to *Leishmania donovani* (Ld; acc. no. A2CIL3), *Leishmania major* (Lm; acc. no. Q4Q3K1), *Trypanosoma cruzi* (Tc; acc. no. Q1WBU6), *Leuconostoc mesenteroides* (Lmes; acc. no. P11411) and *Homo sapiens* (human; acc. no. P11413). Strictly conserved residues are highlighted with black background, while highly similar residues, with similar physicochemical properties in at least four out of the five sequences are shown in bold letters. The secondary structure elements of *Ld*G6PD with bound NADP(H) (PDBID: 7ZHU) are displayed above the sequence alignment. The magenta coloured box marks the N-terminal domain (M1-K49). Yellow coloured boxes indicate important cysteines (C56, C61, C94, C138). Red delta marks the mutation site of the truncated *Ld*G6PD$^{60\text{-}562}$ and the red asterisk the mutated residue C138 of the *Ld*G6PD^{C138S} point mutant. The structure-based multiple sequence alignment was performed using the program ESPript 3.0 [1].

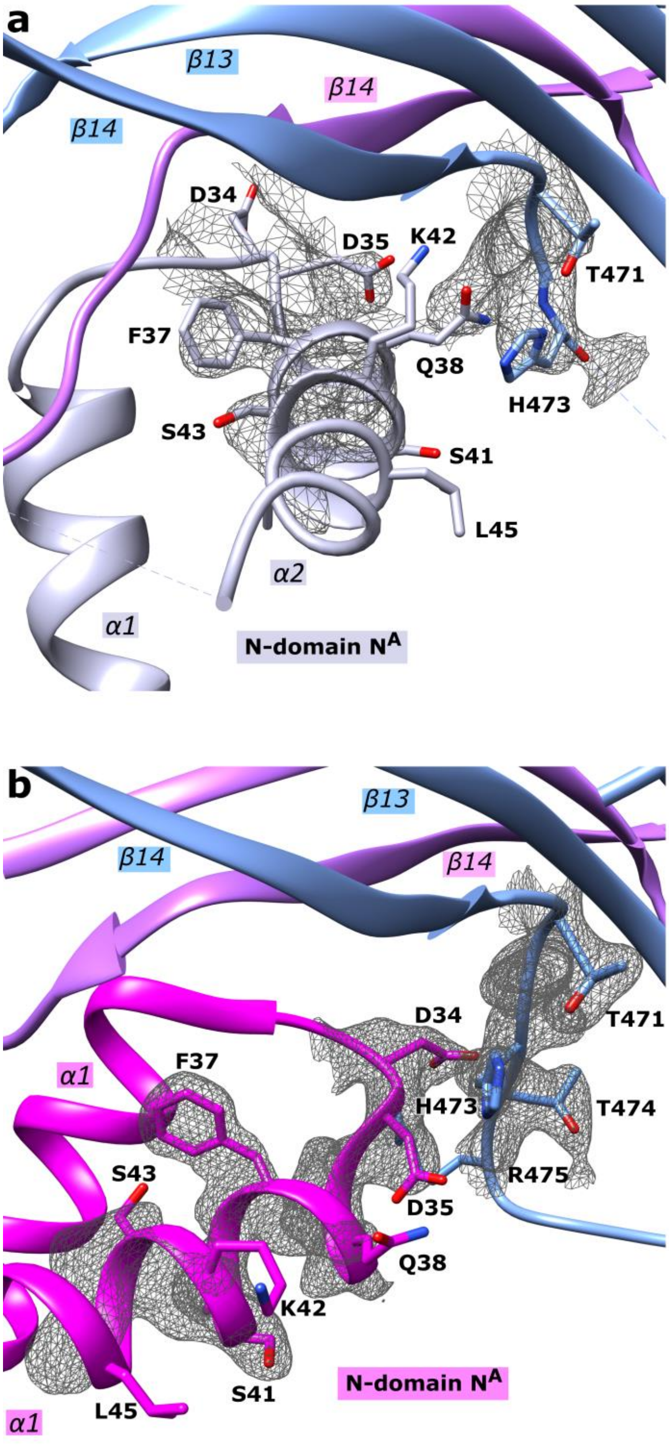

Fig. S4 Substrate-induced shift of the N-domains. The dimers of the apo and the NADP(H) complexed structures are shown in the same orientation (PDBIDs: 7ZHT, 7ZHU). The β-α domain of subunits A or B are colored light purple and light blue, respectively. Electron density map (F_O-F_C polder omit map) contoured at 2.5 σ around residues D34-L45 from N-domain A and residues T471-R475 of the loop region are shown in black. **a** apo structure: N-domain A (N^A) is colored grey. **b** NADP(H) complexed structure: N-domain A (N^A) is colored magenta. Important residues are shown in stick models. Respective α-helices and β-sheets are numbered.

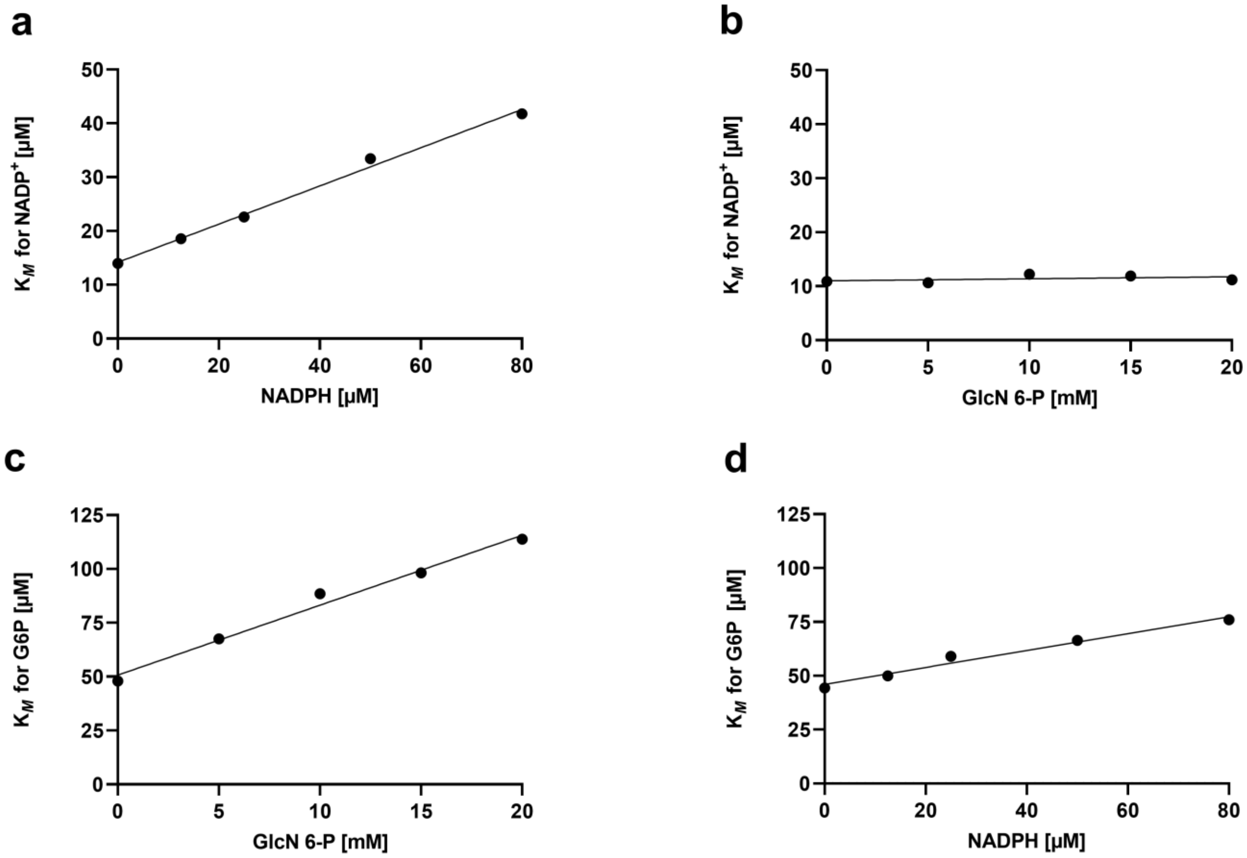

Fig. S5 Inhibition of *Ld*G6PD by the product inhibitor NADPH and the dead-end inhibitor glucosamine 6-phosphate. NADPH **a, d** and glucosamine 6-phosphate (GlcN 6-P) **b, c** were titrated at various constant concentrations of the the substrate G6P or cosubstrate $NADP^+$. The apparent K_M values of $NADP^+$ and G6P, respectively, are plotted against the various concentrations of the inhibitors NADPH and GlcN 6-P. Increasing concentrations of NADPH increased the K_M for $NADP^+$ **a** and for G6P **d** and increasing concentrations of GlcN 6-P increased the K_M for G6P **c**, but not for $NADP^+$ **b**. Representative graphs from three independent replications are shown.

Table S2 Codonoptimized nucleotid sequence

***Ld*G6PD wt**	5’ATGAGTGAAGAACAGAGCCATGCCGATCAGGATGCATATGTTGCAGATGTGGATGGCATTCTG<br>GATGTGCTGCGTGCACAGGTTCTGGAACGTAAACCGGATGATATTTTTCAGTTTATTAGCAAAAG<br>CGCCCTGAGCCTGCAGAAAGATCGCGGTGCAGAAAGCTGTGATCGTATTAATTGTAAAGTTAAG<br>GACGAACAGAAGAGCCGCGCACTGACCATTATTGTTTTTGGTGCAAGCGGTGACCTGGCCAAAA<br>AGAAAACCTTTCCGGCACTGTTTGATCTGTATTGCGGTGGTCTGCTGCCGCCGGAAGTGAATATT<br>ATTGGTTATGCACGTACCAAAGTTGATGATGTTGAAAAATGGAAACACGAAACCCTGATGAAAT<br>ATTTTAGTAATCTGAGTGAGCGCGGTTGCCATGCCGAAGATTTTCTGAAACATATTAGCTATTTC<br>TGCGGCGCATATGATAGTGTTGATGATTTTAAACGCCTGGATGCCGTGATTCGCGAAAAAGAAA<br>ATGCCTTTAAAGGCCCGGAAAAAGGTGGCAATCGTCTGTTTTATCTGGCCCTGCCGCCGAGCGTG<br>TTTGCAAGTGTGTGTGAAAGCATTCATAAAGGCGCCATGCCGCAGGAAGTTGGTGGTTGGGTTC<br>GTGTTATTATTGAAAAACCGTTTGGTCGCGATACCAAAAGTAGTGCAGAACTGAGCCAGGCACT<br>GGAACCGTTTTTCGATGAAAGTCAGCTGTATCGTATTGATCATTATCTGGGTAAAGAAATGGTGC<br>AGAATATTATTACCACCCGTTTTGCCAATCGCATTTTTAGCGCCGTGTGGAATGCCAGCAATATT<br>GCCTGCGTGCAGATTACCTTTAAAGAAACCATTGGCACCGAAGGCCGTGGCGGTTATTTTGATA<br>ATATTGGTATTATCCGCGACGTTATGCAGAATCATCTGACCCAGATTCTGGCACTGCTGGCAATG<br>GAAAAACCGCGTAGTCTGGATGCAGAATGTATTCGTGATGAAAAAGTTAGTGTTCTGAAATGTA<br>TCGAACCGATTACCAAAGAAAATTGTGTTCTGGGCCAGTATACCGCCAGCGCCGATGGCAGCAT<br>TCCGGGTTATCTGGAAGATGTTACCGTGCCGGAAGGCAGTACCTGCCCGACCTTTGCCGTTATGC<br>GCCTGAATATTAATAATGATCGCTGGGCCGGTGTTCCGTTTATTCTGAAAGCCGGTAAAGCCGTG<br>GAACAGAAATATGTGGCAATTCGTATTCAGTTTCGCGATGAAGTGCATCCGTATGGTGAAGCCA<br>CCCAGCGTAATGAACTGGTTATTCGCGCACAGCCGAGTGAAGCAATGTATGTTAAAATTACCAC<br>CAAAGTGCCGGGTCTGAGTGGTGACCTGCGCCAGACCCATCAGACCGAACTGGATCTGACCTAT<br>CATACCCGCTATGATGTTCGTCTGCCGGATGCATATGAAAGCCTGATTAATGATGCCCTGCTGGG<br>CAATAGCACCAATTTTGTTCGTAAAGATGAACTGGATGTTGCATGGCGCATTTTTACCCCGCTGC<br>TGCATCAGATTGATAGCGGCGAAATTAAGCCGATTCCGTATCAGGCAGGTACCCGTGGTCCGAA<br>AGAAGCAGATGAGTTTATTGCAAATAACGGCTTTAAACATCAGAAAGGCTATCATTGGCTGCCG<br>AGCAATAAGCTGTAA-3’
***Lm*G6PD wt**	5’ATGAGCGAAGAACAGAGTCATGCCGATCAGGATGCATACGTTGCCGATGTGGATGGCATTCTG<br>GATGTTCTGCGTGCAGAAGTTCTGGAACGCAAACCGGATGATGTTTTTCAGTTTATTAGTCAGAG<br>TGCCCTGAGTCTGCAGAAAGATCGCGGCGCCGAAAGTTGCGATCGTATTAATTGCAAAGTGACC<br>GATGAACAGAAAAGCCGTGCCCTGACCATTATTGTGTTTGGCGCCAGCGGTGACCTGGCCAAAA<br>AGAAAACCTTTCCGGCACTGTTTGATCTGTATTGTGGCGGCCTGCTGCCGCCGGAAGTGAATGTT<br>ATTGGTTATGCACGTACCAAAGTTGATGATGCAGAACGCTGGAAACGCGAAACCCTGATGACCT<br>ATTTTAGTAATGTGCCGGAACGCGCCTGTCATGCAGAAGATTTTCTGAAACATATTAGTTACTTC<br>TGCGGTAGTTATGATAAAGTGGATGATTTTAAGCGCCTGGATGCCGTGATTCGCGAAAAAGAAA<br>ATGCCTTTAAAGGTCCGGAAAAAGGTGGCAATCGCCTGTTTTATCTGGCACTGCCGCCGAGTGTT<br>TTTGCAAGCGTGTGCGAAAGCATTCATAAAGGCGCCATGCCGCAGGAAGTGGGCGGCTGGGCAC<br>GTGTTATTATTGAAAAACCGTTTGGTCGTGATACCAAAAGTAGCGCAGAACTGAGTCGTGCCCTG<br>GAACCGTTTTTCGATGAAAGTCAGCTGTATCGTATTGATCATTATCTGGGTAAAGAAATGGTTCA<br>GAATATTATTACCACCCGTTTTGCAAATCGCATTTTTAGCGCAGTGTGGAATAGTAATAATATTG<br>CCTGCGTGCAGATTACCTTTAAAGAAACCATTGGCACCGAAGGCCGTGGTGGTTATTTTGATGGT<br>ATTGGTATTATTCGTGACGTGATGCAGAATCATCTGACCCAGATTCTGGCACTGCTGGCCATGGA<br>AAAACCGCGCAGCCTGGATGCCGAATGTATTCGTGATGAAAAAGTGAGTGTGCTGAAATGCATT<br>GAACCGATTACCAAAGAAAATTGTGTGCTGGGCCAGTATACCGCCAGCGCAGATGGCAGTATTC<br>CGGGCTATCAGGAAGATGTTACCGTTCCGGAAGGTAGTACCTGCCCGACCTTTGCCGTTATGCGT<br>CTGAATATTAATAATGATCGCTGGGCCGGTGTGCCGTTTATTCTGAAAGCAGGTAAAGCAGTTGA<br>ACAGAAATATGTTGCAATTCGTATTCAGTTTAAGGATGAAGTTCATCCGTATGGCGAAGCCACCC<br>AGCGCAATGAACTGGTTATTCGCGCCCAGCCGAGCGAAGCCATGTATGTTAAAATTACCACCAA<br>AGTTCCGGGTCTGAGTGGCGATCTGCGTCAGACCCATCAGACCGAACTGGATCTGACCTATCAT<br>ACCCGTTATGATGTTCGTCTGCCGGATGCATACGAAAGCCTGATTAATGATGCCCTGCTGGGTAA<br>TAGTACCAATTTTGTGCGTAAAGATGAACTGGATGTGGCATGGCGTATTTTTACCCCGCTGCTGC<br>ATCAGATTGATTGTGGTGAAATTAAGCCGATTCCGTATCAGGCAGGCACCCGCGGCCCGAAAGA<br>AGCAGATGAGTTTATTACCAATAACGGTTTTAAGCATCAGAAAGGTTATCAGTGGCTGCCGAGC<br>AATAAGCTGTAA-3'

Table S3 Oligonucleotide primers used for mutagenesis of *L. donovani* G6PD

**Mutant**	**Sequence**	
***Ld*G6PD^{C138S}**	Sense	5'-GAGCGCGGTTCCCATGCCGAA -3'
	Antisense	5'-ACTCAGATTACTAAAATATTTCATCAGGGTTTCG-3'
***ΔLd*G6PD$^{60\text{-}562}$**	Sense	5'-ATATGGATCCAATTGTAAAGTTAAGGACGAAC-3'
	Antisense	5'-ATATAAGCTTTTACAGCTTATTGCTCGGC-3'

Supplementary References

1. Robert, X. & Gouet, P. Deciphering key features in protein structures with the new ENDscript server. *Nucleic Acids Res.* **42,** W320-4; 10.1093/nar/gku316 (2014).
